# Eccentric contraction-induced strength loss in dystrophin-deficient muscle: Preparations, protocols, and mechanisms

**DOI:** 10.1085/jgp.202213208

**Published:** 2023-01-18

**Authors:** Leonit Kiriaev, Cory W. Baumann, Angus Lindsay

**Affiliations:** 1 Muscle Research Group, Murdoch Children’s Research Institute, Parkville, Victoria, Australia; 2 School of Medicine, Western Sydney University, Campbelltown, New South Wales, Australia; 3 Ohio Musculoskeletal and Neurological Institute (OMNI), Ohio University, Athens, OH, USA; 4 Department of Biomedical Sciences, Ohio University, Athens, OH, USA; 5 Institute for Physical Activity and Nutrition, School of Exercise and Nutrition Sciences, Deakin University, Geelong, Victoria, Australia

## Abstract

The absence of dystrophin hypersensitizes skeletal muscle of lower and higher vertebrates to eccentric contraction (ECC)-induced strength loss. Loss of strength can be accompanied by transient and reversible alterations to sarcolemmal excitability and disruption, triad dysfunction, and aberrations in calcium kinetics and reactive oxygen species production. The degree of ECC-induced strength loss, however, appears dependent on several extrinsic and intrinsic factors such as vertebrate model, skeletal muscle preparation (in vivo, in situ, or ex vivo), skeletal muscle hierarchy (single fiber versus whole muscle and permeabilized versus intact), strength production, fiber branching, age, and genetic background, among others. Consistent findings across research groups show that dystrophin-deficient fast(er)-twitch muscle is hypersensitive to ECCs relative to wildtype muscle, but because preparations are highly variable and sensitivity to ECCs are used repeatedly to determine efficacy of many preclinical treatments, it is critical to evaluate the impact of skeletal muscle preparations on sensitivity to ECC-induced strength loss in dystrophin-deficient skeletal muscle. Here, we review and discuss variations in skeletal muscle preparations to evaluate the factors responsible for variations and discrepancies between research groups. We further highlight that dystrophin-deficiency, or loss of the dystrophin–glycoprotein complex in skeletal muscle, is not a prerequisite for accelerated strength loss-induced by ECCs.

## Teaser

Kiriaev et al. discuss how preparations, protocols, and factors impact sensitivity to ECC of dystrophin-deficient skeletal muscle.

## Introduction

Skeletal muscle dystrophin is a cytoskeletal protein that links the intracellular actin cytoskeleton to the extracellular matrix through the dystrophin–glycoprotein complex (DGC; Ervasti, 2007; Rybakova et al., 2000; Ervasti and Campbell, 1993). Dystrophin provides stability to the sarcolemma (Ramaswamy et al., 2011; Straub et al., 1997; Mokri and Engel, 1975), facilitates mechanotransduction (Kumar et al., 2004; Ramirez et al., 2022), and is critical for cellular signaling (Brenman et al., 1995; Rando, 2001; Allen et al., 2016; Janke et al., 2013). When dystrophin is nonfunctional or absent in humans, skeletal muscle suffers from a severe pathology that causes Duchenne muscular dystrophy (DMD). Dystrophin-deficient skeletal muscle cells of laboratory animals are susceptible to accelerated strength loss (Kiriaev et al., 2022; Lindsay et al., 2021; Lindsay et al., 2019; Baumann et al., 2020; Nelson et al., 2018; Head et al., 1992; Moens et al., 1993), sarcolemmal disruption (Olthoff et al., 2018; Whitehead et al., 2006; Lindsay et al., 2020), disruption in neuromuscular junction morphology (Pratt et al., 2015; 2013), loss of excitability (Roy et al., 2016; Call et al., 2013; Baumann et al., 2020), disrupted Ca^2+^ transport (Bellinger et al., 2009; Gervásio et al., 2008), and generation of reactive oxygen species (ROS; Whitehead et al., 2010) during and following eccentric contractions (ECCs). These phenotypic manifestations of dystrophin-deficient skeletal muscle to ECCs provide optimal differentiation from wildtype muscle, ensuring proposed treatments for DMD can be appropriately evaluated.

Due to the robust phenotype of dystrophin-deficient skeletal muscle to ECCs, research laboratories have established and even developed their own preparations and protocols to understand the mechanisms of strength loss or to test preclinical therapies for DMD. However, it has become apparent that the degree of strength loss across research groups can vary significantly (Lindsay et al., 2020). The variability in loss of strength in dystrophin-deficient skeletal muscle can be attributed to several extrinsic factors, such as animal model (mouse, rat, canine, or fish), skeletal muscle preparation (in vivo, in situ, or ex vivo), ECC protocol (number, length, duration, and stimulation of contractions), and muscle hierarchy (single fiber, whole muscle, or muscle compartment). Intrinsic factors have also been shown to associate with the degree of sensitivity to ECCs in dystrophin-deficient skeletal muscle, such as muscle strength, fiber type (utrophin and cytoplasmic actin content), fiber branching, age, and background of the animal model. These preparations and protocols, while serving the laboratory’s individual purpose, cast doubt and confusion on the mechanisms of ECC-induced strength loss in dystrophin-deficient muscle, which has potential implications on evaluating the efficacy of DMD treatments. Therefore, this review will cross-examine the extrinsic and intrinsic factors impacting strength loss in dystrophin-deficient skeletal muscle to ECCs, summarize the mechanisms of strength loss and evaluate the efficacy of genetic editing techniques to restore dystrophin expression and limit strength loss induced by ECCs. This will ensure a concise understanding of the ECC assay is appropriately transcribed for future study designs and discrepancies observed in the published literature can be explained. This review defines strength loss as force (or torque) deficits that occur during or immediately after an ECC protocol.

## Duchenne muscular dystrophy

DMD is a fatal X-linked neuromuscular disease affecting 1 in 5,000 boys (Crisafulli et al., 2020). DMD is caused by loss of function mutations to the gene encoding dystrophin (Hoffman et al., 1987). The loss of expression or function of dystrophin renders skeletal muscle cells susceptible to injury and degeneration (Vohra et al., 2015; Mokri and Engel, 1975). Consistent skeletal muscle degeneration and regeneration cause skeletal muscle fibers to become centrally nucleated, highly variable in size and shape, atrophy, weaken, and lose their regenerative capacity (Bell and Conen, 1968; Mokri and Engel, 1975). This pathological appearance of skeletal muscle is driven by the consistent degeneration that is accompanied by infiltration of immune cells for restorative purposes and the replacement of contractile tissue with fat and fibrotic deposits (Rosenberg et al., 2015). Over time, this process forces patients with DMD to become non-ambulatory, seek ventilatory support later in life, and die from cardiorespiratory failure in their 20s (Nigro et al, 1990; Landfeldt et al, 2020). The absence or nonfunctionality of dystrophin drives this pathological hallmark of DMD, and it is thought that the loss of sarcolemmal stability and structure provided by dystrophin predisposes it to damage and strength loss during stresses endured throughout muscle contractions. More specifically, it is inferred, based on preclinical data, that skeletal muscle ECCs contribute to DMD pathophysiology.

## Eccentric contractions

ECCs (muscle lengthening while activated) are a part of normal physiological functioning and serve as shock absorbers to decelerate during landing tasks or to precisely deal with high external loading such as those found in downhill skiing (Vogt and Hoppeler, 2014) and walking (Hu et al., 2020) This type of contraction incorporates contractile and series elements to evoke the greatest force production from skeletal muscle. In fact, a maximal ECC can produce considerably more force than that of an isometric or concentric contraction (Lindstedt et al., 2001). It is generally accepted that ECCs cause skeletal muscle damage or injury because of their intrinsic capacity to produce high levels of stress (force generated per fiber). Functionally, even in healthy human and rodent muscle, ECCs result in immediate and prolonged reductions in strength (Lindsay et al., 2021; Byrne et al., 2001; Warren et al., 2001).

As we will outline in this review, hypersensitivity to ECC-induced strength loss in dystrophin-deficient skeletal muscle (versus healthy muscle) has been observed or theorized to occur within several sites and processes of muscle contraction and mechanotransduction (Baumann et al., 2022; Kumar et al., 2004). Furthermore, the mechanisms of ECC-induced strength loss appear to be dependent on the physiological preparations and contractile protocols, in addition to various extrinsic and intrinsic factors. Regardless, the published literature clearly indicates that dystrophin plays a critical role in maintaining optimal force production during and after ECCs in fast-twitch muscle. The following sections will summarize the required knowledge researchers must appreciate when studying ECC-induced strength loss in dystrophin-deficient skeletal muscle.

## DMD animal models

The extensive number of laboratory-generated (gene edited) or naturally occurring animal models of DMD (>60; McGreevy et al., 2015) provides genetic variation to encapsulate the high variability in genetic mutations observed in patients with DMD (Bladen et al., 2015). It further allows the assessment of low and high vertebrates for optimal clinical translation because eccentrically damaging muscles from patients with DMD is unethical and likely to exacerbate skeletal muscle pathology. The *mdx* mouse, which does not express full-length dystrophin (Dp427, referring to the full isoform size present in striated muscle) due to a nonsense point mutation in exon 23 of the gene encoding dystrophin, is the most studied animal model in DMD research. Skeletal muscle of *mdx* mice is highly sensitive to ECC as first evidenced in the extensor digitorum longus (EDL) and diaphragm muscles (Petrof et al., 1993; Head et al., 1992; Moens et al., 1993). Depending on the preparation of muscles from *mdx* mice, strength loss can range from 0 to 95% (Consolino and Brooks, 2004; Muir et al., 2016; Tinsley et al., 1998; Liu et al., 2005; Zanou et al., 2009; Gehrig et al., 2008; Martin et al., 2009; Chan et al., 2007; Petrof et al., 1993; Lindsay et al., 2020; Whitehead et al., 2006; Lindsay et al., 2019; Nelson et al., 2018; Moens et al., 1993). Several other mouse models of dystrophin-deficiency also manifest a similar sensitivity to ECC, which will be discussed in depth in a later section.

**Figure 1.**
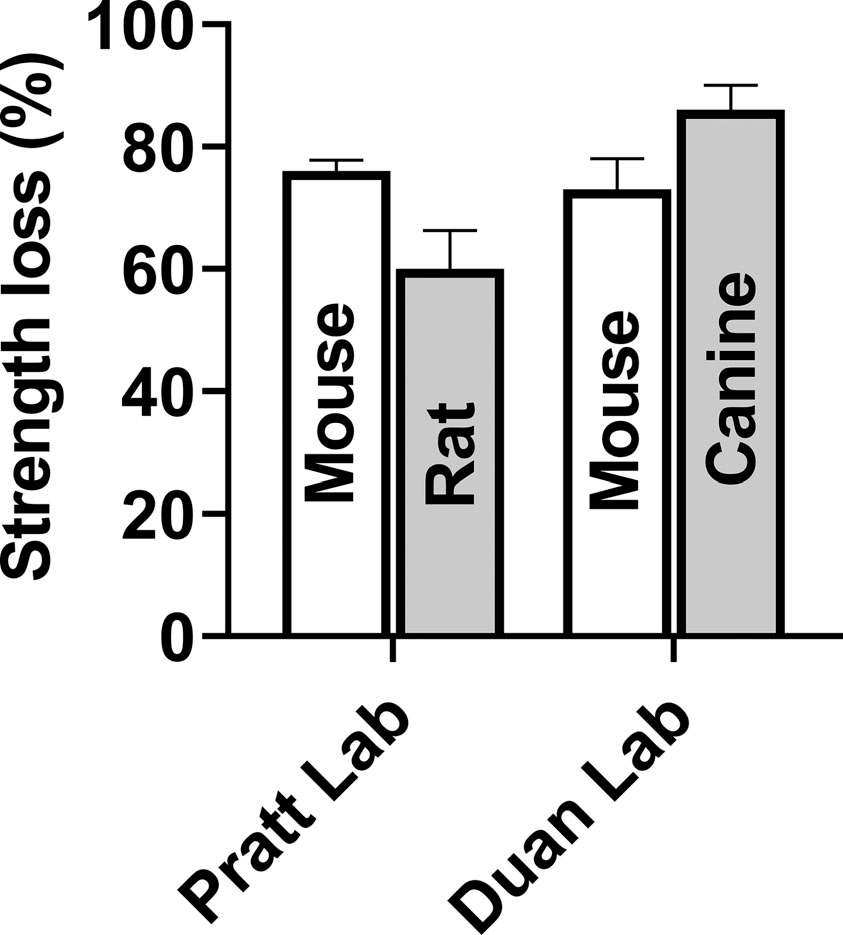
**Strength loss in dystrophin-deficient skeletal muscle of mice (*mdx*), rats (Dmd-KO), and canines (mixed genetic background of golden retriever, Labrador retriever, Welsh corgi, and beagle) following a single bout of eccentric contractions.** Skeletal muscle in the Pratt laboratory were quadriceps and exposed to the same in situ eccentric contraction protocol (15 eccentric contractions through a 40–110° arc of motion spaced 1 min apart). In the Duan laboratory preparations, the mouse EDL muscle was exposed to 10 ex vivo eccentric contractions spaced 2 min apart (10% L_o_ at 0.5 L_o_/s for 200 ms) and the canine extensor carpi ulnaris muscle was exposed to 10 in situ eccentric contractions spaced 2 min apart (5% L_o_ at ∼7–8 mm/s). Data are mean ± SEM and adapted from Pratt et al. (2013), Iyer et al. (2020), Liu et al. (2005), and Yang et al. (2012). Note the universal loss of strength for all dystrophin-deficient mammalian models, but the variation between and within species and laboratories that is likely associated with muscle type, mammalian age, and eccentric contraction protocol.

Transitioning toward higher vertebrates, the rat model of DMD (Larcher et al., 2014) provides a 10× body size, scalable model relative to the mouse. Although the rat model of DMD has been available since 2014, to the best of our knowledge, only one study has investigated the effect of ECCs on strength (Iyer et al., 2020). Here, the tibialis anterior (TA) and quadriceps muscle of DMD rats lost ∼60–70% strength compared to ∼20–40% of wildtype rats. These values are like that described for mouse models, although a comparison is challenging given a single study was completed in vivo with a specific protocol on two muscles. However, when comparing strength loss in mice and rats from the same research group and under the same conditions, it appears *mdx* (68–76%) and wildtype mice (22–39%) lose approximately the same level of strength (Fig. 1; Sanchez et al., 2017; Pratt et al., 2013). Therefore, it appears, independent of rodent size, that strength loss is comparable in skeletal muscle when dystrophin is abolished.

Comparing the sensitivity of higher vertebrates to ECCs in mice or rats deficient in dystrophin is challenging, given few laboratories possess the technical capability to study across species and even fewer studies have assessed sensitivity to ECCs in dystrophin-deficient skeletal muscle of larger vertebrates. When skeletal muscles of dystrophin-deficient canines are assessed for their sensitivity to ECCs, consideration must be given to the overall function and load/work of these muscles compared to smaller vertebrates. For example, the hindlimb compartment of rodents and dogs carries 45–60% and 30% of body mass, respectively (Williams et al., 2019; Itoi et al., 2021). Dystrophin-deficient skeletal muscle of canines are hypersensitive to ECCs, losing between 63 and 88% of strength compared to 8–36% for control canines (Yang et al., 2012; Tegeler et al., 2010). While these values appear like mice and rats, closer inspection of the preparations indicates differences within canine studies compared to rodent studies. For example, canines of different species (golden retriever, Labrador retriever, Welsh corgi, and beagle), muscle groups (tibiotarsal flexor muscles and extensor carpi ulnaris), muscle preparation (in situ versus in vivo), and ECC protocols (muscle length change, stimulation duration, stimulation frequency, number of contractions, and time between contractions) are different between studies. Additionally, when a laboratory does have the technical and financial capacity to examine sensitivity to ECC-induced strength loss across species, different protocols and preparations are used (Yang et al., 2012; Liu et al., 2005), making it difficult to determine whether vertebrate level is a factor influencing degree of sensitivity (Fig. 1).

The sensitivity of the dystrophin-deficient zebrafish to ECCs makes species comparison even more challenging, primarily because of the dearth of published studies and the variability in protocol preparation. The dystrophin-deficient zebrafish, or larvae, do not have individual muscles measured, but rather it is a whole-body preparation. When subjected to an ECC protocol, dystrophin-deficient larvae lose 50–95% strength compared to <20% for unaffected larvae (Widrick et al., 2016). Once again, strength loss is comparable to higher order vertebrates, but a closer inspection of the protocols identifies dramatic differences, even within the same group of researchers. For example, zebrafish larvae were subjected to a single ECC (200-μs pulses at 300 Hz for 20 ms; lengthened 5 or 10% of optimal preparation length at a velocity of 1.0 optimal preparation length/s) while EDL and diaphragm muscle of *mdx* mice were subjected to five ECCs (200-μs pulse at 200–300 Hz for 150–250 ms; lengthened 20% of fiber length [L_f_] at 1.5 L_f_/s; Widrick et al., 2011). To properly compare species, a relatively standardized protocol at minimum should be investigated.

It appears that independent of vertebrate order, dystrophin-deficiency results in significant levels of strength loss in multiple skeletal muscles following ECCs. These levels of strength loss appear dependent on the preparation and protocol, with a species-specific design implemented between and within research groups. Due to the high variability among preparations and protocols, and the lack of difference in the published literature between vertebrates in their sensitivity to ECCs, the remainder of this review will focus primarily on studies investigating mouse models of DMD, with a particular focus on the C57BL/10-*mdx*, unless otherwise stated.

## Factors impacting strength loss-induced by ECCs

### ECC preparations

**Figure 2.**
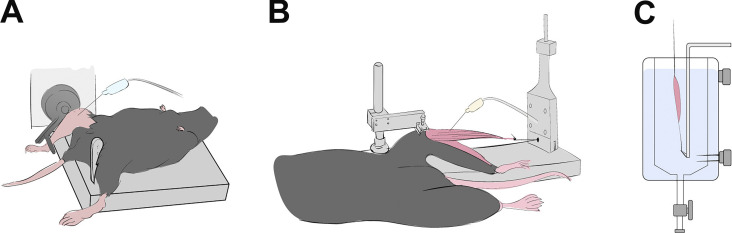
**Drawings illustrating examples of preparations used to investigate ECC-induced strength loss in dystrophin-deficient *mdx* mouse studies. (A–C)** Preparations assessing function of muscle compartments in vivo (A), in situ preparations measuring intact whole muscle function while maintaining nerve and blood supply (B), and ex vivo preparations where the whole muscle excised from the animal (C).

There is tremendous variability in the methods to measure susceptibility to contraction-induced strength loss of *mdx* muscle and still more variability in the interpretation of these results. Strength loss immediately after isolated ECCs can range from 0 to 95% in *mdx* skeletal muscle, and this finding has been consistently reported in muscles in situ and ex vivo, as well as entire muscles groups in vivo (Fig. 2).

**In vivo (**Fig. 2 A**).** Depending on muscle groups investigated, the posterior crural muscles of the *mdx* mouse can lose between 38 and 72% (Blaauw et al., 2010; Blaauw et al., 2008; Call et al., 2011; Call and Lowe, 2016) of starting torque following bouts of ECCs compared to 62–73% reported in anterior crural muscles with variations attributable to differences between in vivo preparations (Belanto et al., 2014; Novotny et al., 2014; Belanto et al., 2016; Call and Lowe, 2016; Baumann et al., 2022; Call et al., 2013; Nelson et al., 2018; Call et al., 2011). Although not often detailed in the research methodology, in vivo preparations can have the animal on its side or in a supine position affecting the nerve access, location of electrodes and working range of rodent ankles during locomotion (Charles et al., 2018). Fixation of the tibia and angle of electrode insertion should enable the ankle to be free to rotate through a full range of motion during contraction (Iyer et al., 2016). Deviations in magnitude of rotation and therefore length change have been shown in Lindsay et al. (2020) to tune the sensitivity of *mdx* anterior crural muscles to ECC-induced torque loss. With a greater excursion, muscle lengthening will be longer and fewer ECCs are required to elicit maximal strength loss.

One of the most technical parts of the in vivo preparation is the optimal placement of electrodes and stimulation parameters inhibiting electrical current “bleeding” out of the area of interest into other muscle groups. Early preparations in *mdx* mice by Sacco et al. (1992) isolated and stimulated the cut sciatic nerve before it branches out to the common peroneal and tibial nerves to investigate anterior crural muscles (labeled as TA) during in vivo contraction. The resultant co-contracture elicited only an 8% difference in strength after employing a vigorous eccentric injury protocol (240 contractions spaced 5 s apart at 100 degrees of movement). Due to the heavier mass of the collective plantar-flexors relative to dorsi-flexors, a co-contraction would result in both an ECC of the dorsi-flexors as well as a concentric contraction of the plantar-flexors affecting the potential strength loss in *mdx* muscles (Hakim et al., 2005). Preparations removing nerve innervation to opposing muscles (Call and Lowe, 2016; Blaauw et al., 2010; Blaauw et al., 2008) and the insertion of wire/cuff implants (Baumann et al., 2021; Call et al., 2011; Baumann et al., 2020; Baumann et al., 2022) have been implemented to reduce these instances in the *mdx* mouse, with the majority of investigators placing electrodes alongside the nerve of interest (Novotny et al., 2014; Call et al., 2013; Belanto et al., 2014; Nelson et al., 2018; Belanto et al., 2016; Lindsay et al., 2020).

**In situ (**Fig. 2 B**).** In dystrophin-deficient *mdx* literature, primarily the TA and EDL muscles have been investigated for contraction-induced strength loss in situ reporting a 35–95% variation across studies (Brooks, 1998; Dellorusso et al., 2001; Terry et al., 2014; Spinazzola et al., 2015; Godfrey et al., 2015; Potter et al., 2021; Roy et al., 2016; Bellinger et al., 2009). During in situ experiments where the distal tendon is surgically cut and released from attachment, it is important to release the muscle entirely from its origin to avoid lateral force transmission from nearby muscles (Ramaswamy et al., 2011), a detail often omitted that could explain the variation in strength loss following eccentric injury in *mdx* studies. Efforts to minimize lateral contraction of the entire hindlimb have resulted in variations between electrode placement, nerve preparation, and stimulation parameters.

TA studies on *mdx* mice use various methods of surgical exposure (Hakim et al., 2011; Potter et al., 2021) and crushing of the sciatic nerve proximally (Spinazzola et al., 2015; Roy et al., 2016), whereas common practice in other laboratory groups places electrodes directly on the peroneal nerve for stimulation (Brooks, 1998; Terry et al., 2014; Dellorusso et al., 2001; Godfrey et al., 2015). When comparing ECC stimulation parameters across these dystrophin-deficient studies using the same equipment setup for TA mechanics, stimulation frequency varied between 50 and 250 Hz. It is possible that these differences in electrode preparations have influenced the sensitivity of nerves to stimulation and therefore the recruitment of muscle fibers for supramaximal stimulation. In turn, this can explain the variation in specific force reported in TA muscles from *mdx* (13–21 N/g) and control (20–35 N/g) mice across these in situ studies. Consequently, this may impact the extent to which fibers are recruited to produce maximal force during muscle ECCs and indirectly impact the strength loss in ECC studies (Lindsay et al., 2020).

Bellinger et al. (2009) investigated the contractile physiology of the *mdx* EDL ex vivo through indirectly stimulating the belly of the TA laying superficial to the EDL muscle. The preparation at the time was novel compared to traditional ex vivo EDL preparations where electrodes are placed along the peroneal nerve for stimulation (Anderson et al., 1988; Brooks, 1998) and shown to be effective at producing muscle force without compromising the nerve itself. The *mdx* EDL muscle from Bellinger et al. (2009) produced 35 N/g of specific force compared to the 18–30 N/g seen in traditional EDL in situ preparations from *mdx* mice of the same age (Brooks, 1998; Anderson et al., 1988). Furthermore, the *mdx* EDL lost up to ∼60% of its starting force after a single ECC (15% of optimal length [L_o_]) compared to the ∼70% reported in situ by Brooks (1998) induced by 10 ECCs (20% L_f_) performed with 10 s rest between contractions. Potentially, the higher starting strength of the dystrophin-deficient EDL muscles in the Bellinger preparations impacted the sensitivity it has to ECC-induced strength loss, which will be discussed in depth in a later section of this review.

**Figure 3.**
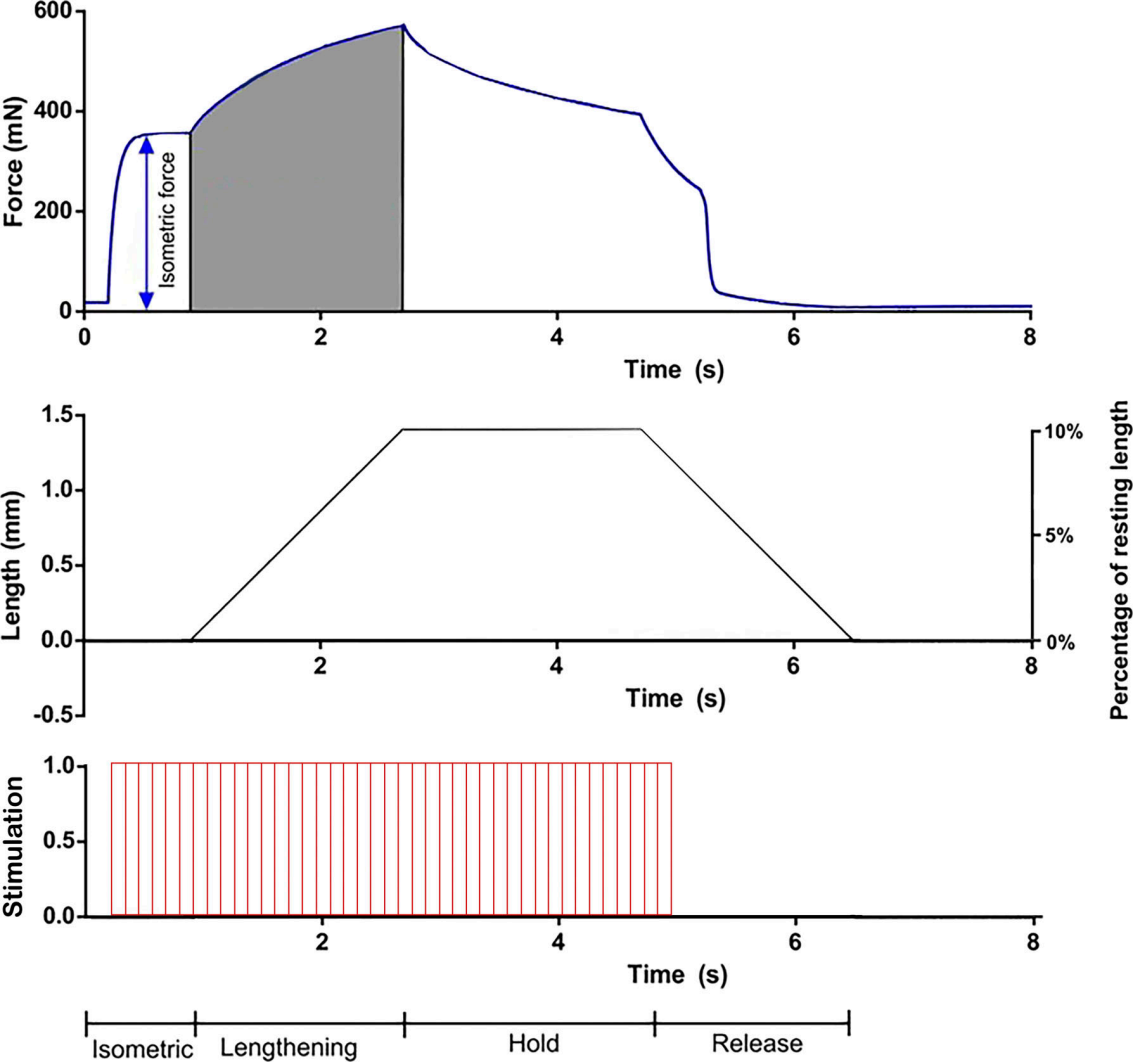
**Typical ECC force (top), length (middle), and electric stimulation (bottom) trace obtained during a single 10% ECC performed on an EDL muscle ex vivo.** Data from Kiriaev et al. (2018). The y-axis for the middle trace represents the distance lengthened amounting to 10% of the muscle length away from resting length (14 mm). Electrical stimulation conducted at frequency 125 Hz and duration 1 ms starts t = 0.2 s, at t = 1 s the muscle is stretched at velocity 1 mm/s until it is 10% longer than its resting length, held at this length for 2 s, then returned at the same rate to its original length. Stimulation is stopped at t = 5 s. The protocol is repeated successively during preparations with adequate rest intervals. Highlighted grey area under the force trace is the total work done during the eccentric lengthening portion of the protocol. Variations in this setup exist between laboratories as several parameters can be manipulated for each muscle preparation. An example of an in vivo ECC protocol performed on the plantar-flexors can be viewed in Call and Lowe (2016).

**Ex vivo (in vitro,** Fig. 2 C**).** Most ex vivo (also known as in vitro) preparations start lengthening contractions from L_o_ preceding the eccentric lengthening with an isometric stimulation, as depicted in Fig. 3. Alternative preparations by the Lowe group in *mdx* mice prefer to precede eccentric lengthening with a passive shortening to 50% of the total length change before applying the ECC. The goal of implementing such a protocol is to prevent overstretching the muscle and keep excursion length within physiological ranges in vivo. Arguably, preceding the eccentric lengthening with a passive shortening in such a way would reduce the strain applied beyond L_o_ by half and can impact the degree of strength loss induced by ECCs. However, the Lowe group has consistently reported ∼80–90% strength loss in EDL muscles from *mdx* mice that have undergone 10 ECCs (10% strain pre-shortened) spaced 3 min apart (McCourt et al., 2018; Lindsay et al., 2018; Olthoff et al., 2018; Nelson et al., 2018; Belanto et al., 2014). These values are comparable to other studies (Martin et al., 2009; Miura et al., 2009) using similar ECC protocols (10 contractions at 10% strain with 2–3 min rest) implemented at L_o_ to investigate EDL muscles from *mdx* mice, reporting ∼70–90% strength loss following ECCs. Thus, implementing passive shortening preceding lengthening contractions does not appear to impact strength loss compared to ex vivo studies starting lengthening from L_o_.

Ex vivo muscle preparations including the EDL (with fast contractile properties), the soleus (with slow muscle properties) and diaphragm (exhibiting the most pathological progression of muscular dystrophy; Stedman et al., 1991) make up a large majority of the dystrophin-deficient literature investigating vulnerability to ECC-induced strength loss. When these studies are separated based upon muscles of interest, the dystrophin-deficient slow-twitch soleus has consistently reported little to no strength loss (0–15%) following ECCs in *mdx* mice (Consolino and Brooks, 2004; Gehrig et al., 2008; Kiriaev et al., 2021a; Head et al., 1992; Lindsay et al., 2019; Moens et al., 1993). In contrast, fast-twitch EDL from the *mdx* mouse has reported between 10 and 95% strength loss across multiple studies employing varying strains of muscle lengthening (5–30% of L_o_; Capogrosso et al., 2017; Muir et al., 2016; Kiriaev et al., 2022; Kiriaev et al., 2018; Call and Lowe, 2016; Novotny et al., 2014; Tinsley et al., 1998; Liu et al., 2005; Chan et al., 2007; Zanou et al., 2009; Petrof et al., 1993; Whitehead et al., 2006; Miura et al., 2009; Belanto et al., 2014; Consolino and Brooks, 2004; Martin et al., 2009; Nelson et al., 2018; Moens et al., 1993; Belanto et al., 2016; McCourt et al., 2018; Olthoff et al., 2018; Kiriaev et al., 2021a).

In dystrophin-deficient EDL and soleus studies, several researchers have employed a shorter length change multiple times in quick succession instead of fewer lengthening contractions at a higher strain (Kiriaev et al., 2021b; Chan et al., 2007; Lindsay et al., 2020) to achieve strength loss in dystrophin-deficient muscles. Utilizing ECC protocols that allow minimal rest between repeated contractions increases the duty cycle (contraction duration/interval between contractions) and risks introducing aspects of fatigue-induced loss of strength when strength loss is measured immediately afterwards. This is particularly evident in Muir et al. (2016) and Zanou et al. (2009) investigating ECC-induced strength loss in EDL muscles from 2–4-mo-old *mdx* mice reporting a severe 88–95% strength loss after 6–7 ECCs spaced only 10 s apart. When compared to other age-matched dystrophin-deficient mice from Whitehead et al. (2006) and Tinsley et al. (1998), similar eccentric strains spaced 30–60 s apart elicited only a 55–65% strength drop in *mdx* EDL muscles. To minimize the fatigue introduced during repeated contractions, often research groups prefer to employ a single lengthening contraction (between 5 and 60% L_f_) for inducing strength loss (of up to 70% in *mdx* EDL) to focus on only the mechanical factors that contribute to initiation of injury (Lynch et al., 2000; Consolino and Brooks, 2004; Gehrig et al., 2008).

To compare the extent of stretch for ECC preparations in situ and ex vivo to physiologic excursions seen in vivo, we must correct muscle length change during contraction to its corresponding fiber length change when stretched. These correction values are unique to each muscle due to differences in muscle length, fiber length, and fiber pennation angle. Commonly used fiber correction ratios for EDL (0.44), soleus (0.71), and TA (0.61) are reported in Brooks and Faulkner (1988) and Burkholder et al. (1994). Recent modeling of muscle fiber length changes (fiber excursions) in mouse and human hindlimbs across one gait cycle by Hu et al. (2017) has allowed a physiological comparison to in vivo movements for each of these preparations. Considering the range of mouse ankle joint excursion is between 40 degrees dorsiflexion and 20 degrees plantarflexion, the range of movement and fiber excursion reported for each of the ECC preparations above are well within the physiological limits of the mouse hindlimb during a movement gait cycle.

Similar to EDL preparations, ex vivo studies investigating diaphragm muscle strips in dystrophin-deficient *mdx* mice exhibit between 13 and 57% strength loss after ECC-induced strength loss (Morine et al., 2010; Moorwood et al., 2013; Gehrig et al., 2008; Petrof et al., 1993). Interestingly, most of these lengthening protocols were conducted at 10% L_o_ undergoing similar contractile conditions suggesting the variation in preparations between groups are not protocol related. In diaphragm muscle strips isolated for ex vivo studies, the width, thickness, and consequent mass vary unpredictably among animals at the discretion of the investigator excising them. Dissection methods for *mdx* diaphragm preparations used by the Barton group are described in detail by Moorwood et al. (2013). During contractile measurements, the group found that strips of diaphragm that were wider than 5 mm begin to fold on themselves during contractions compromising force generation because the central tendon tie is located at only one point. Constraints on optimal diaphragm strip size (2–4 mm wide) have thus been set by Barton and Lynch laboratories (Lynch et al., 1997), so that the strip is large enough to contain only a small portion of dissection-damaged fibers but small enough to not fold over during contraction. Both the width of the damaged region as well as the width of the entire preparation are important factors to control in dystrophin-deficient diaphragm studies and can help explain some of the variation in strength loss following ECCs reported between groups (13–57%).

In general, the smaller the desired organ scale to study (such as the peroneus longus, lumbrical, and flexor digitorum brevis), the more cumbersome and tedious the ECC preparations become (Claflin and Brooks, 2008; Yeung et al., 2003; Lindsay et al., 2019). This holds particularly true for mechanical isolation procedures in small mouse muscles with researchers opting for enzymatic digestion to produce high yields of intact fibers from *mdx* mice that may not be suitable for ECC studies because the procedure can potentially render the T-system voltage sensors dysfunctional (Lamb and Stephenson, 2018). Single fiber procedures enable assessment of susceptibility to ECCs on only one fiber type, which allow force and other changes (ionic, metabolic) associated with ECC-induced strength loss to be unequivocally correlated. These preparations also allow rapid application of extracellular drugs, ions, and metabolites prior to contraction-induced damage as well as during recovery (compared to whole muscles which require time to diffuse across gradients). Both Yeung et al. (2005) and Yeung et al. (2003), and later Whitehead et al. (2010), have utilized these aspects of intact single fiber preparations to investigate the effects of pharmacological inhibition of stretch-activated channels and NADPH oxidase (stretch-induced ROS production) on [Ca^2+^]_i_ influx and force following a series of ECCs.

Skinned fiber preparations eliminate the permeability barrier whilst leaving the sarcoplasmic reticulum (SR) intact allowing the study of myofibrillar properties such as Ca^2+^ release and uptake during contraction. Several *mdx* mouse studies have used chemically skinned fiber preparations to study contraction-induced strength loss in the gastrocnemius (Blaauw et al., 2010; Blaauw et al., 2008), EDL (Lynch et al., 2000), and TA (Roy et al., 2016). Interestingly, studies employing ECCs on chemically skinned *mdx* fibers did not report a significant difference compared to control counterparts and is inconsistent with observations reported in whole muscle preparations. This finding is particularly evident in Blaauw et al. (2008) and Blaauw et al. (2010), who showed chemically skinned fibers taken from an *mdx* gastrocnemius muscle that was exposed to ECCs in vivo produced 28% less isometric force compared to wildtype controls. When chemically skinned fibers were isolated from an uninjured *gastrocnemius* and exposed to ECCs ex vivo, no significant differences in strength loss were seen between *mdx* and wildtype fibers. The observation that there is no difference in force deficits of single permeabilized fibers from *mdx* and control muscles in these studies provides indirect evidence that the permeabilized “skinning” process both mechanically or chemically removes the surface membrane eliminating any protection conferred by dystrophin and the DGC by disruption of the DGC and cytoskeletal linkages.

### Extrinsic factors

The accurate measurement of muscle force producing capacity and strength loss during ECC-induced strength loss in *mdx* mice are dependent upon a multitude of external factors (extrinsic factors) influencing each preparation.

**Oxygen diffusion.** Muscle size is a limiting factor for ex vivo assays due to concerns surrounding the development of anoxic cores in larger muscles during muscle contraction (Bonen et al., 1994). Multiple laboratory groups have set a muscle size limit (∼20 mg) for ex vivo preparations after reporting diminished force production and stability in larger muscles (Hakim et al., 2013; Call and Lowe, 2016; Moorwood et al., 2013). For muscles such as the TA that can range between 70 and 100 mg in the *mdx* mouse (Kiriaev et al., 2021b), the in situ approach is preferred as normal blood supply remains undisturbed and hypoxia-associated artifacts are avoided (Hakim et al., 2013). Diaphragm strips conversely do not suffer from the same complications (anoxic core) because they are thin enough to have prolonged viability in the perfused organ bath (Moorwood et al., 2013). Oxygen diffusion modeling by Barclay (2005) concluded ex vivo preparations remain viable at duty cycles below 0.1 at 35°C and 0.3 at 25°C. This means dystrophin-deficient *mdx* mouse studies employing high ECC duty cycles (Muir et al., 2016; Zanou et al., 2009) spaced 10 s apart make strength loss interpretations from ECCs indistinguishable from anoxic damage resulting in an excessive degree of strength loss reported in these *mdx* studies.

**Temperature.** In studies investigating muscle susceptibility to ECC-induced strength loss, the preparations are highly temperature sensitive (Warren et al., 2001). The majority of *mdx* ex vivo preparations investigating susceptibility to ECC-induced strength loss have been conducted between 20 and 25°C with only a few exceptions between 27 and 30°C (Capogrosso et al., 2017; Liu et al., 2005; Martin et al., 2009). The disparity in temperatures (10°C) reported across preparations brings into question whether the variability in strength loss reported in these *mdx* muscles have been potentiated to some degree by temperature-dependent excitation–contraction coupling failure or decreased oxygen perfusion. For permeabilized single fiber preparations, sub-physiological experimental temperatures such as 15°C are commonly used to improve the reproducibility of mechanical measurements. It is also possible to generate valid data at higher temperatures (requiring a higher ATP turnover) as long as the effects of temperature on solution properties (Ca^2+^ concentration, pH, etc.) are taken into consideration (Roche et al., 2015).

**Solutions used for preparations.** Most groups investigating ECC-induced strength loss in *mdx* use a physiology salt solution with negligible variations in ionic composition across groups (Krebs-ringer/mammalian ringers/ringers) buffered with either carbogen or oxygen. The Lowe laboratory has included insulin and branched chained amino acids in buffered Krebs and similarly the Head laboratory has added fetal bovine serum to improve the viability of ex vivo ECC preparations for up to 2 h (Olthoff et al., 2018; Kiriaev et al., 2021b) by suppressing an increase in basal rate of glucose and amino acid uptake. A similar concept applies for fully activated permeabilized fiber preparations that are undergoing eccentric stretches. The addition of creatine phosphate is important for buffering the intermyofibrillar ATP and ADP fluctuations that would be associated with contractile activity. During *mdx* ECC experiments where fibers are fully activated and undergoing high turnover rates of ATP, creatine kinase is added in some laboratories (Roche et al., 2015; Lynch et al., 2000) but not others (Blaauw et al., 2008; Blaauw et al., 2010) to supplement the endogenous creatine kinase that remains bound to the fiber. These small variations in solution constituents between *mdx* studies can, therefore, affect the viability of preparations following ECC and their strength generation capacity.

**Anesthetic influence on preparations.** Anesthetic agents can interfere with several sites to alter muscle contractile mechanics including the neuromuscular junction (Gergis et al., 1972), excitation–contraction coupling (Marwaha, 1980), and contractile proteins (Leuwenkroon-Strosberg et al., 1973). In *mdx* studies, the Lowe, Lynch, and Head laboratories have primarily used sodium pentobarbital and isoflurane to sedate mice prior to muscle extraction, whereas the Barton and Duan laboratories have favored ketamine, xylazine, and acepromazine. These anesthetics have demonstrated differential effects in skeletal muscle contractile function (Ingalls et al., 1996) and elicited varying effects to Ca^2+^ sensitivity (Kunst et al., 1999). The variation in anesthetics used between groups may explain the disparity in strength loss observed following ECCs in dystrophin-deficient mice studies.

**Consistency between laboratories.** Accurate measurement of muscle force producing capacity is dependent upon several factors including surgical dissection (which varies based on technique and experience) and use of accurate force recording equipment (such as the Aurora complete muscle systems popular in recent publications) to ensure muscles are adequately stimulated to recruit all motor units within the muscle and adequate muscle perfusion. The evaluation of muscle strength loss in response to ECC in vivo and ex vivo should ensure preparations produce consistent maximum forces relative to muscle mass and age across laboratories. If there is a significant discrepancy between these values, there are deficiencies in the preparations or equipment and if any of these extrinsic parameters are overlooked, the measurement of the muscle’s true functional capacity will be inaccurate and therefore the strength loss from ECCs. A review by Grounds et al. (2008) of standard operating procedures for preclinical studies in the *mdx* mouse critically evaluates the multitude of factors that can influence variation between similar experiments across different laboratories. The review stresses the importance of implementing a basic standardization of experimental approaches setting a benchmark for *mdx* and C57BL/10 mice and implementing minimal parameters to assist with comparisons between laboratories.

### Intrinsic factors

An intrinsic factor can be defined as the morphological, physiological, and molecular properties of a skeletal muscle that can be influenced by age and genetic background. Intrinsic factors of this nature have recently been investigated to explain the variability in strength loss in dystrophin-deficient mouse skeletal muscle following ECCs.

**Figure 4.**
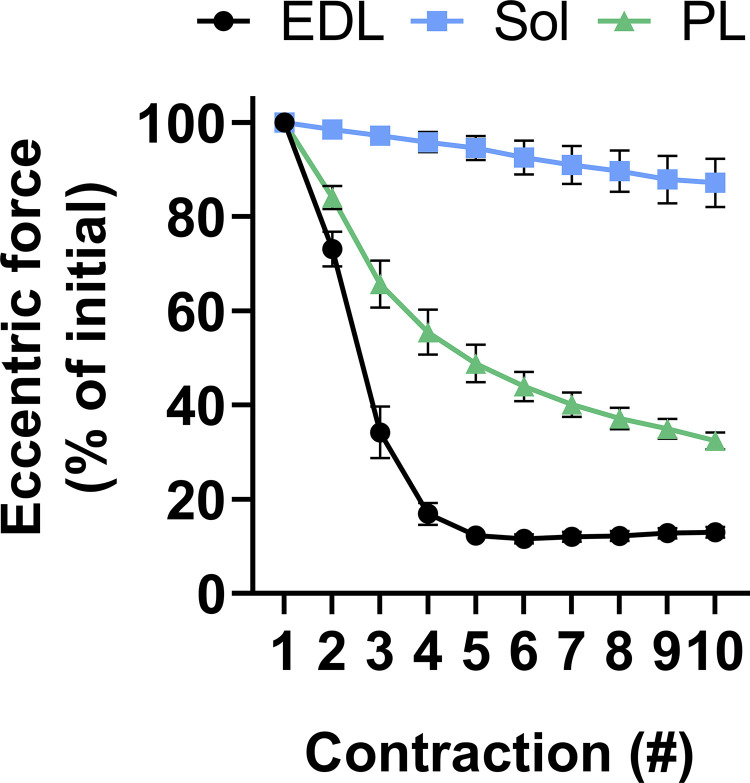
**Eccentric contraction force tracings of the EDL (fast-twitch), soleus (Sol; slow-twitch), and peroneus longus (PL; intermediate fiber type) muscle of *mdx* mice subjected to an identical ex vivo protocol (10 contractions with a 10% length change at 0.5 L**_**o**_**/s).** Data are mean ± SEM and adapted from Lindsay et al. (2019).

**Muscle fiber characteristics.** A study by Lindsay et al. (2019) was designed to measure differences in strength loss between three different *mdx* skeletal muscles subjected to identical ex vivo protocols (10 contractions with a 10% length change at 0.5 L_o_/s). The EDL, soleus, and peroneus longus muscles of *mdx* mice presented with similar pathological hallmarks of dystrophin-deficiency but with high variability in their sensitivity to strength loss following ECCs. After five contractions, the soleus muscle had lost 5% strength, the peroneus longus 50%, and the EDL 90% (Fig. 4). Comparison of intrinsic factors between these three skeletal muscles determined that the greater the density of type IIb fibers, the faster and greater the loss of strength (R^2^ = 0.847). Utrophin expression levels closely follow type IIb fibers as a major predictor of strength loss in *mdx* skeletal muscle to ECCs (R^2^ = 0.815). Soleus and peroneus longus muscles had ∼2.5- and ∼1.5-fold greater utrophin expression relative to EDL, respectively. Utrophin overexpression in *mdx* skeletal muscle attenuates sensitivity to ECCs ex vivo and partially in vivo (Belanto et al., 2014; Tinsley et al., 1998). Moreover, abolishing utrophin expression in *mdx* mice further sensitizes multiple skeletal muscles to ECCs (Lindsay et al., 2019), suggesting utrophin has a major impact on this phenotype. Other major predictors of *mdx* skeletal muscle sensitivity to ECCs include type IIa fibers (R^2^ = 0.753) and type I fibers (R^2^ = 0.626). It is generally accepted that slow-twitch fibers are inherently less susceptible to ECC-induced strength loss when compared with fast-twitch fibers in both clinical (Barthel et al., 2021) and preclinical studies (Lindsay et al., 2021; Kiriaev et al., 2021a). Fast-twitch fibers develop greater relative force, and this would be expected to impart greater physical stresses on the dystrophin-deficient membrane. Ultrastructural observations show slow-twitch fibers have wider Z-lines and higher amounts of protein connecting the actin filaments of adjacent sarcomeres compared with fast-twitch fibers (Luther, 2009). These wider Z-lines in the slower fibers are suggested to provide a structural response to stress make them resistant to ECC damage.

Interestingly, two ROS-related predictors were discovered that moderately correlated with strength loss sensitivity: cytoplasmic γ-actin and cytoplasmic β-actin (R^2^ = 0.626–0.631). Overexpression of either cytoplasmic isoform in *mdx* mice provides protection from ECC-induced strength loss by blocking ROS production through NADPH oxidase II due to an oxidation-sensitive cysteine 272 unique to cytoplasmic actins (Baltgalvis et al., 2011; Olthoff et al., 2018). These data suggest ROS is a factor influencing susceptibility to ECCs, which has been confirmed by Olthoff et al. (2018), who showed that knockout of the antioxidant peroxiredoxin in *mdx* skeletal muscle increases sensitivity to ECCs but only when the protocol intensity is reduced. This suggests that the protocol influences sensitivity to ECCs of *mdx* muscle through a ROS-dependent mechanism.

**Muscle strength.** Lindsay et al. (2019) showed that the strength produced by the skeletal muscles of *mdx* mice was not a predictor of strength loss-induced by ECCs (R^2^ = 0.208). However, in another study by Lindsay et al. (2020), where they used the EDL muscle for ex vivo testing, the anterior hindlimb muscles for in vivo testing (EDL, TA, and extensor hallucis longus) and manipulated the mechanical properties of the contraction (contraction duration, contraction velocity, and length/angle change), strength became a moderate predictor ex vivo (R^2^ = 0.310) and in vivo (R^2^ = 0.400). These data suggest that strength of a dystrophin-deficient muscle influences sensitivity to ECCs in *mdx* mice, which closely resembles evidence in wildtype mice, where peak muscle tension is the dominant predictor in ECC-induced injury in rat soleus muscle (Warren et al., 1993). To partially support strength as a predictor of sensitivity to ECCs in *mdx* skeletal muscle, there is a moderate association between strength loss and work completed during ECCs (R^2^ = 0.542–0.563). Here, the muscle or muscle groups remain identical, the parameters of the contraction were changed to influence displacement, and this was multiplied by force of the contraction. The greater the displacement of the muscle(s) and the more force it produced, the faster the rate and overall loss of strength in *mdx* muscle in response to ECCs (Lindsay et al., 2020).

**Fiber branching.** A branched fiber is a result of consistent muscle degeneration due to dystrophin-deficiency that becomes hypersensitized to ECCs (Head et al., 1990). The question posed by researchers is whether branching or dystrophin-deficiency is the driver of ECC-induced strength loss. The Head laboratory, with minor protocol variations (number of contractions, length of the contraction, and contraction velocity), has shown that fiber branching is associated with strength loss in fast-twitch EDL muscles (Kiriaev et al., 2018; Chan et al., 2007; Kiriaev et al., 2021b). In contrast, slow-twitch soleus muscle(s) from *mdx* mice that also contain an increasing number and complexity of branched fibers (Kiriaev et al., 2021a) appear to be spared from ECC-induced strength loss (Gumerson et al., 2010; Moens et al., 1993; Lindsay et al., 2019; Kiriaev et al., 2021a). These data suggest fiber branching partially explains sensitivity to ECCs in *mdx* skeletal muscle.

**Age.** Skeletal muscle pathology becomes increasingly severe in aged *mdx* mice (Massopust et al., 2020; Pastoret and Sebille, 1995; Chamberlain et al., 2007), which may impact ECC sensitivity. Kiriaev et al. have completed two longitudinal studies on slow-twitch soleus muscle (Kiriaev et al., 2021a) and fast-twitch EDL muscle (Kiriaev et al., 2021b) where the ECC protocol and preparation were identical. Soleus muscle, independent of age, did not change in its sensitivity to ECCs, although pathology became more severe. In contrast, aged *mdx* EDL muscle became more sensitive to ECCs, where 9–22-mo-old muscle lost 80–90% strength in one contraction. While this evidence clearly indicates sensitivity changes with age, the protocol appears to be so intense (20% length change, unspecified duration, or velocity) that differences in sensitivity cannot be determined between older groups. Utilizing a shorter length change with alterations in contraction duration should provide an optimal foundation to evaluate age as a predictor of sensitivity of *mdx* skeletal muscle to ECCs.

**Figure 5.**
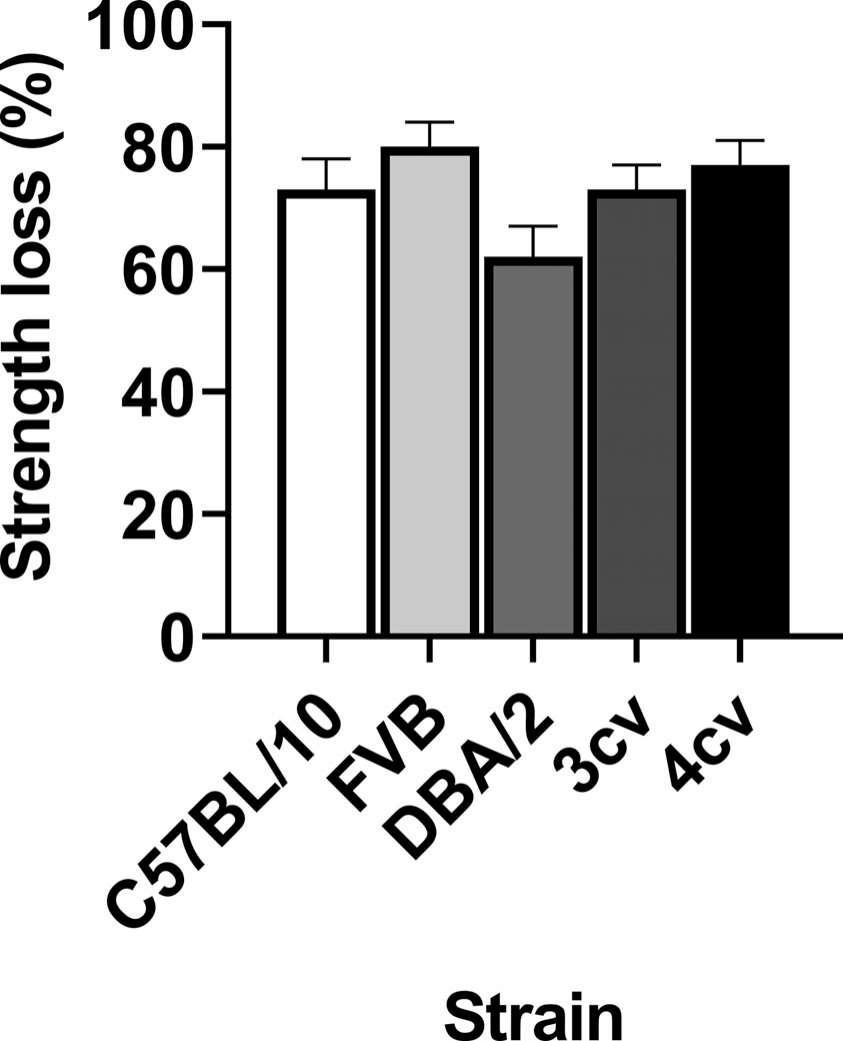
**Effect of genetic background on strength loss induced by 10 ex vivo eccentric contractions (10% L**_**o**_ **at the speed of 0.5 L**_**o**_**/s) in dystrophin-deficient EDL muscle of *mdx* mice.** Each dystrophin-deficient strain was tested in the Duan laboratory using an identical protocol. Data are mean ± SEM and adapted from Liu et al. (2005), Wasala et al. (2015), Hakim et al. (2017), Li et al. (2008), and Zhang et al. (2013). All animals are ∼5–6 mo of age. Note the variability in strength loss between genetic strains.

**Genetic background.** The *mdx* mouse, which does not express endogenous dystrophin in skeletal muscle, has been crossed onto several background strains, including C57BL/10, C57BL/6, DBA/2, albino, C3H, FVB, and 129/Sv. We acknowledge that the *mdx* mouse has been crossed onto immune-deficient strains as well, but this likely provides greater complexity when comparing against standard backgrounds in relation to ECC sensitivity. When the *mdx* mutation is crossed onto these various background strains or generated through a point mutation, skeletal muscle pathophysiology can remain unchanged or become less or more pathological relative to the C57BL/10 background (van Putten et al., 2019; Hammers et al., 2020; Fukada et al., 2010; Wasala et al., 2015; Stenina et al., 2013; Calyjur et al., 2016). When challenged by ECCs with an identical protocol ex vivo, skeletal muscle of FVB-*mdx* mice loses 70–85% strength compared to 65–80% strength of C57BL/10-*mdx* mice at ∼6 mo of age (Wasala et al., 2015; Liu et al., 2005). When the identical protocol challenges DBA/2-*mdx* skeletal muscle, 50–70% strength is lost (Hakim et al., 2017), suggesting at least under identical conditions from the same research group, comparable strength is lost in *mdx* mice independent of background (Fig. 5). This evidence in the DBA/2-*mdx* mouse is corroborated by others who show under identical ex vivo conditions, EDL muscle of DBA/2J-*mdx* mice loses strength at the same rate as C57BL/10-*mdx* mice (Nelson et al., 2018). To the best of our knowledge, no study has investigated the sensitivity of skeletal muscle of albino-*mdx*, C3H-*mdx*, or *mdx*^129^ skeletal muscle to ECCs. Similarly, we could not identify any study that had investigated the sensitivity of C57BL/6-*mdx* skeletal muscle to ECCs. However, there are some studies that have assessed the sensitivity of *mdx* skeletal muscle to ECCs and used C57BL/6 mice as controls (Barton et al., 2005; Barton-Davis et al., 1999; Barton et al., 2002; Lou et al., 2012). Based on the information published, we cannot determine what background the *mdx* mice were on, but results indicate 30–60% strength was lost after ECCs. Confounding these results is the high variability in each protocol, where muscle lengthening ranged from 10% of L_o_ to 30% of L_f_ with similar contraction durations. In a separate study by one of the research groups, where the same protocol was tested on isolated EDL muscle of C57BL/10-*mdx* mice, only 16% strength was lost (Barton, 2006). These data suggest that background may have an impact on sensitivity, but it should be interpreted carefully given background strains were never explicitly stated.

Dystrophin-deficiency has been generated in *mdx* mice by various point mutations using the C57BL/6 background: *mdx2cv*, *mdx3cv*, *mdx4cv*, and *mdx5cv*. These *mdx* lines also have variable dystrophinopathy phenotypes (Chapman et al., 1989) including variability in their sensitivity to ECCs (Fig. 5). Sensitivity to ECCs in skeletal muscle of *mdx2cv*, to the best of our knowledge, has not been investigated, but other lines have certainly been tested and under similar conditions. In studies by Duan et al. (Li et al, 2008; Beastrom et al, 2011; Zhang et al, 2013), isolated EDL muscle from 6–9-mo-old mice were lengthened 10% of L_o_ at 0.5 L_o_/s over 200 ms for 10 contractions. After five contractions, *mdx* and *mdx5cv* skeletal muscle lose ∼85% strength compared to 70–85% for *mdx4cv* and 65–80% for *mdx3cv*. It appears that the type of point mutation may influence sensitivity to ECCs in *mdx* mice, but closer inspection of other intrinsic factors reveals subtle differences that could explain the variability. For example, the *mdxcv* strains are on a C57BL/6 background and *mdx3cv* mice express ∼5% of normal dystrophin levels and are relatively stronger than other strains. Therefore, strain variability may impact sensitivity to ECCs in dystrophin-deficient muscle, but it is likely explained by dystrophin expression, which even at extremely low levels can mitigate dystrophinopathy phenotypes in skeletal muscle of mice and patients with a mutation in the gene encoding dystrophin (Godfrey et al., 2015; Hoffman et al., 1989; Wells et al., 1992).

The *mdx*-52 mouse is a model of DMD that lacks dystrophin expression due to exon 52 deletion in the dystrophin gene, has a severe muscle pathology, and is sensitive to ECCs (Vila et al., 2017). Isolated EDL muscle of *mdx*-52 loses ∼50–60% strength after nine contractions when lengthened 10% (L_o_ or L_f_ not specified) at 2 L_f_/s. This loss in strength appears to be lower than that published for *mdx* muscle, and a comparison of strength loss in *mdx* muscle (exon 23 mutation) subjected to a comparable protocol from similar researchers loses 70–80% strength (Heier et al., 2013). Discrepancies that could account for differences between *mdx* and *mdx*-52 skeletal muscle could be the different backgrounds (C57BL/10 versus C57BL/6), age (28 versus 35 d) and unspecified details of the contraction protocol in one of the studies that could impact degree of strength loss (Heier et al., 2013). This is the only study that we are aware of that has determined the sensitivity of the *mdx*-52 mouse to ECCs, but if it is less sensitive to ECCs than the C57BL/10-*mdx* mouse, dystrophin gene mutation may be a factor influencing sensitivity. To complicate the impact of variable dystrophin gene mutations on sensitivity to ECCs is the generation of a transgenic mouse expressing a full-length dystrophin protein harboring a L54R mutation in actin binding domain 1 (McCourt et al., 2018). This mutation causes DMD in humans (Prior et al., 1993) and it represents an alternative model with which to analyze sensitivity to ECCs in a dystrophin-dependent context. When compared to *mdx* mice, isolated EDL muscle from L54R-*mdx* mice shows a comparable drop in strength, solidifying the suggestion that mutation location and corresponding (dys)function of dystrophin is a potential predictor in sensitivity to ECCs.

Evidence suggests intrinsic factors from molecular signatures of skeletal muscle to background strains to dystrophin gene mutations may influence sensitivity to ECC-induced strength loss in the absence of dystrophin. However, it is apparent that most studies used isolated EDL muscle preparations. While this preparation allows for a controlled comparison between research groups and published literature, it does not allow us to determine if these intrinsic factors also influence single muscle preparations ex vivo or whole muscle preparations in vivo.

## Mechanisms of ECC-induced strength loss in dystrophin-deficient muscle

Mechanisms of strength loss due to ECCs in healthy rodent muscle have been reviewed by Warren et al. (2001) and since confirmed by others (Baumann et al., 2022). These publications concluded that immediate reductions in strength in healthy mouse muscle are primarily due to excitation–contraction coupling failure at the level of the DHPR-RyR interface. These studies will not be reviewed in this article, but rather, we will summarize the physiological mechanisms that contribute to ECC-induced strength loss in dystrophin-deficient, fast-twitch muscles. Mechanisms have been categorized based on the sites and/or processes that were shown to be disrupted.

### Plasmalemmal excitability

**Figure 6.**
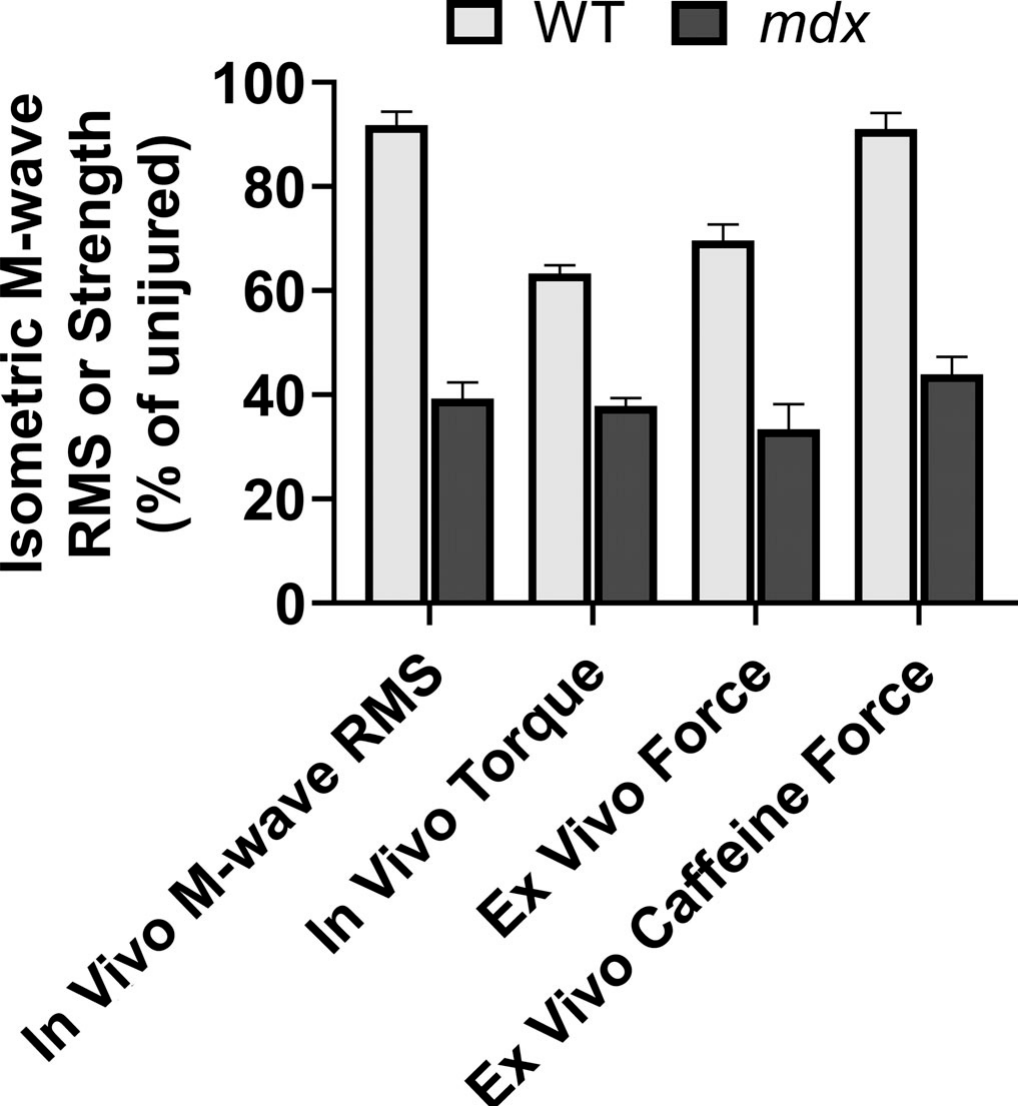
**Deficits in plasmalemmal electrophysiological function and strength parameters in injured muscles of wildtype (WT) and *mdx* mice.** Anterior crural muscles (TA, EDL, and extensor hallucis longus) were injured in vivo and post-injury in vivo anterior crural muscle isometric torque, in vivo TA M-wave root-mean-square (RMS), ex vivo EDL isometric force, and ex vivo EDL caffeine contraction force. Data are mean ± SEM and adapted from Baumann et al. (2020); Baumann et al. (2021); Baumann et al. (2022).

It is well recognized that plasmalemmal excitability measured by M-wave amplitude is reduced during and immediately after *mdx* muscles perform maximal ECCs, and unchanged or only minimally impacted in wildtype muscles (Baumann et al., 2021; Roy et al., 2016; Call et al., 2013; Baumann et al., 2020; Pratt et al., 2013; Fig. 6). Using *mdx* mice implanted with electromyographic electrodes over the TA muscle, it was established that M-wave amplitudes decrease in parallel to that of in vivo eccentric torque of the dorsiflexors (Call et al., 2013; Baumann et al., 2020; Baumann et al., 2021). Indeed, a near perfect linear correlation was observed between M-wave amplitude and torque over 100 maximal ECCs in vivo, with both variables decreasing 60–80%. Logically, a reduction in M-wave amplitude could be due to neuromuscular transmission failure or an inability to generate/conduct an action potential across the plasmalemma. Neuromuscular transmission failure is supported by histological evidence showing that neuromuscular junction morphology becomes increasingly altered in *mdx* quadricep muscle after 15 maximal ECCs in vivo (Pratt et al., 2013; Pratt et al., 2015). An inability to generate/conduct an action potential along the plasmalemma comes from data demonstrating a bout of maximal ECCs depolarized a significant number of *mdx* EDL muscle cells, likely rendering them inexcitable (Call et al., 2013). Moreover, intake of dye into the muscle following ECCs indicate that the plasmalemma is subjected to physical damage (Petrof et al., 1993; Call et al., 2013; Lindsay et al., 2020), which may also contribute to impaired action potential generation/conduction.

By directly stimulating EDL muscles that had previously performed 50 maximal ECCs in vivo, Baumann et al. (2022) reported isometric strength deficits were comparable regardless of nerve or muscle stimulation (Fig. 6). In theory, because high stimulation voltages used for this specific ex vivo preparation activates voltage-gated ion channels on the plasmalemma, which depolarize the muscle fibers, it bypasses neuromuscular transmission and action potential generation/conduction. Therefore, sites and/or processes downstream of the plasmalemma also appear to be disrupted in *mdx* muscle after maximal ECCs (Baumann et al., 2022).

### Ryanodine receptor (RyR) function

RyR monomers interact with numerous ancillary proteins (e.g., calstabin) and are vulnerable to post-translational modifications (e.g., oxidation), both of which influence RyR function (Bellinger et al., 2008; Van Petegem, 2012). RyR function has been shown to both directly and indirectly contribute to ECC-induced strength loss in *mdx* muscle. For instance, preventing calstabin depletion from RyR using a compound that binds to the RyR channel and enhances the binding affinity of calstabin reduced RyR Ca^2+^ leak, and was able to reduce force deficits in *mdx* EDL muscle following a maximal ECC ex vivo (Bellinger et al., 2009). Myricetin, a small molecule modulator that inhibits RyR leak, was also able to provide partial protection from ex vivo strength loss in *mdx* EDL muscle over 10 maximal ECCs (Lindsay et al., 2020). Furthermore, by altering the muscle’s redox balance with N-acetyl cysteine, which theoretically mitigated RyR oxidation and RyR leak, ex vivo ECC-induced strength loss in *mdx* EDL muscle was attenuated (Lindsay et al., 2020; Olthoff et al., 2018).

To determine whether sites and/or processes downstream of the RyRs remain functionally intact, Baumann et al. (2022) assessed ex vivo EDL caffeine contracture forces immediately after 50 maximal ECCs were performed in vivo. Exposing muscle to a high dosage of caffeine promotes Ca^2+^ release from the SR to increase cytosolic Ca^2+^ through activation of the RyRs, thereby bypassing sites proximal to voltage-induced SR Ca^2+^ release (Herrmann-Frank et al., 1999). In injured *mdx* muscles, the reductions in ex vivo isometric and caffeine-induced forces were similar, which was not true for wildtype muscle (Fig. 6). Equivalent reductions in both force outcomes led the authors to suggest mechanisms of strength loss may also be due to SR dysfunction and/or impairment at the myofibrillar apparatus in *mdx* muscle.

### SR function

A key variable for the excitation–contraction coupling process to continue is the reuptake of Ca^2+^ back into the SR through the SR Ca^2+^ ATPase (SERCA). Without replenishment of Ca^2+^ into the SR, voltage-gated Ca^2+^ release through the RyRs will decrease. Data in favor of ECC-induced SR dysfunction occurring in *mdx* skeletal muscle were reported by Mázala et al. (2015). Here, the overexpression of SERCA in *mdx* mice reduced torque deficits after the quadriceps performed 15 maximal ECCs in vivo (Mázala et al., 2015). Lindsay et al. (2020) also demonstrated that using small molecule SERCA activators, in combination with RyR leak inhibitors, attenuated isometric force drop in *mdx* EDL muscles after 10 maximal ECCs ex vivo. In both examples, the observed protection when targeting the SERCA was minor, but significant.

### Cross-bridge formation

Loss of myofibrillar Ca^2+^ sensitivity or disruption to force-generating and/or transmitting elements contributing to the strength loss in *mdx* muscle was obtained using single fiber physiology. Specifically, Ca^2+^ activated force of chemically skinned fibers taken from muscle injured in vivo was significantly lower than uninjured *mdx* fibers (Blaauw et al., 2010), indicating that force could not be restored in injured fibers by bypassing processes at and upstream of SR Ca^2+^ release. In fact, it was reported that after 20 maximal ECCs were performed, in vivo torque and permeabilized fiber force were reduced 38 and 28%, respectively (Blaauw et al., 2010).

Taken together, these data suggest hypersensitivity to ECC-induced strength loss in *mdx* fast-twitch muscle is multifactorial. Indeed, Baumann et al. (2022) recently proposed that these mechanisms may include plasmalemmal electrical generation and/or conduction, SR function, myofibrillar Ca^2+^ sensitivity and components of force-generation and/or transmission. It was also noted that there may be an initiating or catalyzing mechanism that triggers a cascade of disruption up- and/or downstream of the central event. These observations highlight how important selection of the physiological technique (e.g., in vivo, ex vivo) is to the mechanism(s) of strength loss. For instance, if ECCs are performed ex vivo, it must be acknowledged that neuromuscular transmission and plasmalemmal action potential generation/conduction are likely being bypassed, two processes known to be disrupted by in vivo ECCs in *mdx* muscle. Therefore, it is possible that the mechanisms of ECC-induced strength loss in *mdx* muscle may be different when comparing ECCs performed ex vivo to that of in vivo. Another salient point to remember is that all the studies highlighted within this section used maximal ECCs. As we previously reported in (Lindsay et al, 2020), the intensity at which *mdx* muscles perform ECCs impacts their sensitivity to strength loss. Thus, it is entirely possible that the mechanisms of strength loss may differ if *mdx* muscles perform submaximal or maximal ECCs and may vary based on the number of contractions completed (e.g., 15 versus 100).

## Dystrophin restoration strategies

**Figure 7.**
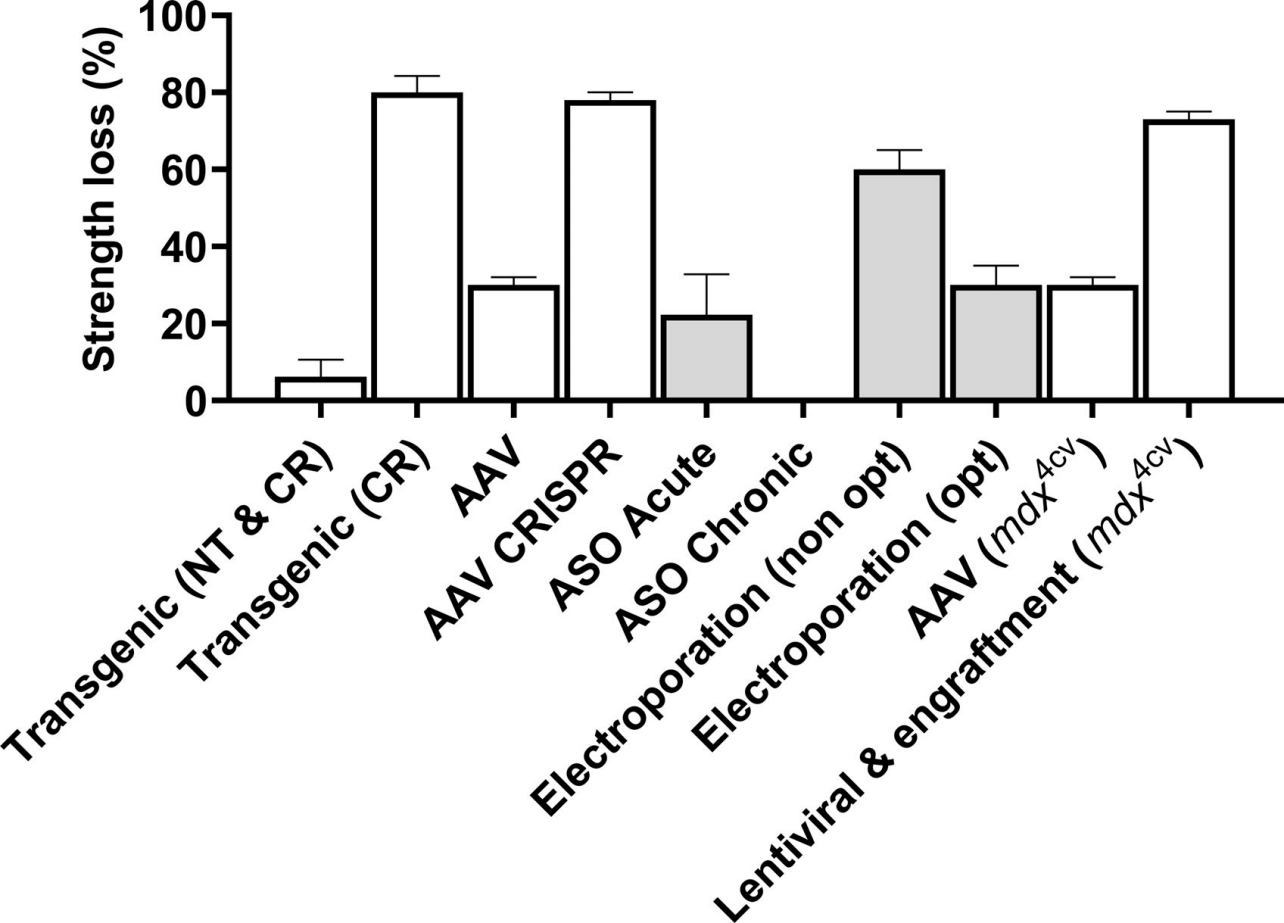
**Strength loss following a series of ECCs observed across various dystrophin restoration strategies employed in C57BL/10-*mdx* (unless specified) by the Chamberlain, Duan, and Wells laboratory groups.** Most studies employed 10 ECCs at 10% L_o_ except for lentiviral engraftment by the Chamberlain laboratory, which employed six ECCs at 30% L_o_. Studies highlighted white were performed in EDL muscles ex vivo whereas studies highlighted in grey were performed in TA muscles in situ. Data are mean ± SEM and adapted from Nelson et al. (2018) (transgenic N-terminal [NT] and cysteine residue [CR]), Liu et al. (2005) (adeno-associated virus [AAV]), Hakim et al. (2018) (AAV CRISPR), Godfrey et al. (2015) (ASO acute and chronic), Foster et al. (2008) (Electroporation non-optimized [OPT] and optimized OPT), Hakim et al. (2020) (AAV *mdx*^4cv^) and Muir et al. (2016) (lentiviral and engraftment *mdx*^4cv^).

Strategies that aim to restore dystrophin expression or function in skeletal muscle use susceptibility to ECC as a functional marker to evaluate efficacy in the C57BL/10-*mdx* mouse. Most studies described here utilize this *mdx* animal unless noted otherwise (Fig. 7).

Transgenic *mdx* mouse lines expressing the mL54R and mL172H mutant full-length dystrophin have been developed to elucidate the pathophysiology of missense dystrophin in vivo. The mL54R line expressing steady-state dystrophin levels of 7–9% presented no differences in susceptibility to ECC-induced strength loss compared with the *mdx* mouse. Homozygous mL172H mice showed a >1.5-fold increase in dystrophin levels compared with hemizygous littermates, which expressed 44% steady-state dystrophin levels and demonstrated significant improvements in ECC-induced strength loss compared with hemizygous mice (McCourt et al., 2018).

The limited packaging size of currently used adeno-associated viral (AAV) vectors only allows for genes smaller than 4.5 kb, therefore precluding the large gene encoding dystrophin. The observation of a severely truncated but highly functional dystrophin prompted the development of mini (or micro)-dystrophins that can restore a certain level of function in preclinical mouse models (Gregorevic et al., 2008; Wang et al., 2009). Most truncations remove portions of the central rod region but retain the N-terminal and cysteine-rich domains because they are critical for binding to actin and the DGC (Norwood et al., 2000). Recently, Nelson et al. (2018) measured the mechanical function across several transgenic mouse lines expressing miniature dystrophins inclusive or exclusive of the C-terminal domain to determine what sections of the dystrophin protein are critical for protection against ECC. All miniaturized dystrophins containing N-terminal and cysteine-rich domains rescued in vivo and ex vivo ECC-induced strength loss compared to *mdx* and Dp116-*mdx* (missing N-terminal domain) mice that lost ∼60–80% of strength after a series of ECCs. Interestingly, Nelson et al. (2018) presented novel evidence that the removal of dystrophin’s R2-3 regions resulted in less protection from ECC relative to other micro- and mini-dystrophins ex vivo.

The re-expression of functional truncated dystrophin was achieved by Roy et al. (2016) using *Dmd* exon 23 skipping strategies through AAV vectors injected into the TA muscles from *mdx* mice. Partial restoration (∼60%) of dystrophin protein expression was confirmed and after 3 wk of vector delivery, the strength loss following ECC was reduced by ∼15% compared to *mdx* muscles injected with saline. Similarly, Hakim et al. (2020) have compared the therapeutic efficacy of AAV micro-dystrophin vectors generated by transient transfection and scalable herpes simplex virus system in *mdx*^4cv^ mice. Both AAV vectors had similar biological potency capable of achieving ∼15–35% reduction in strength loss after ECC at low-medium dosages and complete protection from ECC-injury in EDL muscles at high dosages. Other revolutionary approaches such as AAV-mediated CRISPR editing have been used recently to rescue muscle function in *mdx* mice. Hakim et al. (2018) injected AAV-9 CRISPR vectors to the tail vein of 6-wk-old *mdx* mice and examined dystrophin expression and disease rescue at 18 mo of age. The study found a disproportional guide RNA vector depletion for AAV CRISPR therapy and only at higher vector doses dystrophin expression was restored significantly in skeletal muscle (∼4% expression). In EDL muscles, the modified AAV CRISPR therapy mitigated pathological muscle hypertrophy and rapid strength loss-induced by ECC compared to untreated *mdx* mice. These results suggest that the AAV CRISPR therapy machinery in its early stages still present a unique dosage-based barrier as a dystrophin restoration strategy.

Lentiviral delivery of mini-dystrophin constructs have faced a dosage-based barrier eliciting poor transduction at the sight of injection (Li et al., 2005), prompting most genetic rescue strategies to focus on ex vivo cell therapy. The Chamberlain laboratory has shown that injection of lentiviral vectors containing mini-dystrophin can transduce satellite cells in vivo in young *mdx*^4cv^ mice expressing dystrophin in 20–25% of myofibers for up to 2 yr (Kimura et al., 2010). Subsequent transplantation into immunocompetent *mdx*^4cv^ muscles resulted in EDL muscles with up to 30% regional engraftments showing no significant differences in protection from ECC-induced strength loss compared to saline-injected controls (Muir et al., 2014). To improve the functional impact of in vivo myogenically converted fibroblasts, Muir et al. (2016) later introduced a prosurvival cocktail improving donor cell survival so that EDL muscles contained up to 60% regional engraftments. Following transplantation, the EDL muscles containing mini dystrophin fibers reduced strength loss following ECC by ∼15% compared to untreated *mdx*^4cv^ controls. These studies by the Chamberlain laboratory provide a therapeutically attractive strategy for using patient-accessible fibroblasts cell population to enhance dystrophin expression in DMD; however, there remains a possibility that lentiviral vectors may induce an immune response when utilized at high doses, such as those that would be required for transduction in humans (Chamberlain, 2013).

The injection of naked plasmid DNA was used to perform the first successful example of human dystrophin gene transfer into the *mdx* mouse (Acsadi et al., 1991). Subsequently, several groups have used in vivo electroporation as a physical method of gene delivery to introduce cDNAs to *mdx* muscles. Foster et al. (2008) investigated the impact of codon optimization on micro-dystrophin expression and function in the *mdx* mouse. Plasmid DNA expressing three different micro-dystrophins was injected intramuscularly into the TA muscle of *mdx* mice before commencing electroporation. When these muscles were tested in situ, treatment with both codon optimized and non-optimized AB/R3-R18/CT micro-dystrophins were unable to confer protection from ECC-induced strength loss losing ∼60% of their starting force after 10 ECCs. However, treatment with ΔR4-R23/CT micro-dystrophin provided a significant amount of protection losing only ∼30% of their starting force corresponding to the highest micro-dystrophin expression level of the three cDNA sequences. These results demonstrate that codon optimization of micro-dystrophin can significantly increase expression levels rescuing enough micro-dystrophin to improve susceptibility to ECC strength loss.

Internally truncated dystrophin proteins are partially functional and provide the basis for exon skipping therapy that has placed DMD at the forefront of advances in gene therapy (Aartsma-Rus et al., 2017). Antisense oligonucleotides (ASO) can be used for targeted exon exclusion resulting in the production of an internally deleted but partially functional dystrophin. Godfrey et al. (2015) assessed changes in muscle physiology following treatment of *mdx* mice at 12 wk of age using a phosphorodiamidate morpholino oligomers (PMO)-based ASO to skip *Dmd* exon 23. TA muscles from *mdx* mice measured in situ had significant protection against ECC-induced strength loss when receiving higher doses of PMO-based ASO treatment ∼23.4% (9 mg/kg) and ∼22.3% (12 mg/kg) compared to lower doses ∼54% (3 mg/kg) ∼51% (6 mg/kg) and untreated controls ∼60%. Interestingly, internally deleted dystrophin protein was not detectable in mice given 3 or 6 mg/kg dosages but expressed between 5 and 15% of wildtype dystrophin levels in 9 and 12.5 mg/kg treated mice. When the PMO-based ASO was delivered chronically (10 injections spaced 2 wk apart), full protection from ECC strength loss was achieved. Currently, ASO-mediated modification is the most promising therapeutic intervention for DMD as demonstrated in recent clinical trials (Asher et al., 2020) with a number of them being FDA approved for the treatment of DMD in the USA. Despite this, a major challenge facing the transition of all these therapies to the clinic is how much dystrophin restoration is required for clinical efficacy.

Earlier transgenic studies by Li et al. (2010) have demonstrated that expressing ∼5% of full-length dystrophin significantly enhanced muscle force and prevented muscle necrosis, whereas ASO studies like Godfrey et al. (2015) and Sharp et al. (2011) have managed to show that 15–20% of normal levels of dystrophin were sufficient to prevent strength loss from ECC. A comprehensive study by Van Putten et al. (2012) investigated the effects of low levels of dystrophin expression (3–47%) on mouse muscle function and pathology, concluding dystrophin levels below 15% can improve pathology and performance and levels over 20% are needed to fully protect muscle fibers from ECC-induced strength loss. Importantly, dystrophin restoration studies should standardize the measurement of dystrophin protein when assessing muscle function and implement genetic rescue strategies early before muscle pathogenesis compromises the muscle mechanobiology with age (Kiriaev et al., 2021b).

## Additional considerations

**Figure 8.**
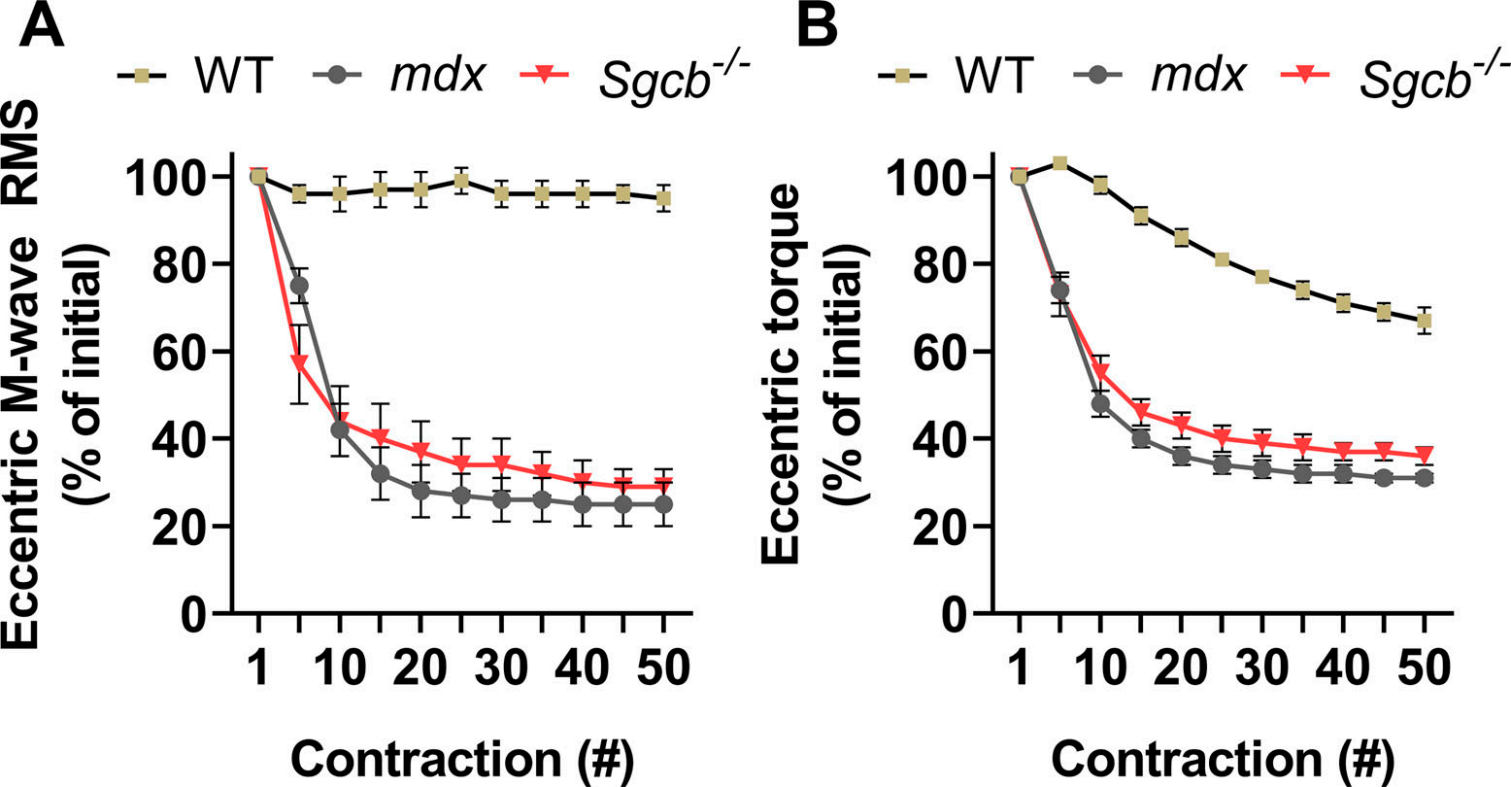
**M-wave root-mean-square (RMS; A) and eccentric torque tracings (B) in wildtype (WT), *****mdx*****, and *****Sgcb***^**−/−**^** (β-sarcoglycan-deficient) mice subjected to an in vivo protocol (50 contractions with a 38° angle change at 2,000°/s).** Eccentric torque was obtained from the anterior crural muscles (TA, EDL, extensor hallucis longus) while M-wave RMS was recorded from the TA. Data are mean ± SEM and adapted from Baumann et al. (2021).

Here and elsewhere, it has been concluded that hypersensitivity to ECC-induced strength loss is linked to dystrophin content. However, we would like to emphasize that there are several caveats to this conclusion. First, as we described in the Muscle fiber characteristics section and depicted in Fig. 4, despite the soleus, peroneus, and EDL muscles all being dystrophin-deficient in the *mdx* mouse, they respond drastically differently to ECCs. In fact, the *mdx* soleus does not appear to be more sensitive to ECCs than that of wildtype soleus muscle (Kiriaev et al., 2021a), even at a length change of 30% (Lindsay et al., 2019). Second, several mouse models that have wildtype levels of dystrophin are known to be *mdx*-like and hypersensitive to ECCs. For example, the *Sgcb*^−/−^ (β-sarcoglycan knockout) mouse expresses wildtype levels of dystrophin at the plasmalemma but lacks the entire sarcoglycan complex (Araishi et al., 1999; Durbeej et al., 2000); yet, it is just as susceptible to ECC-induced strength loss (Belanto et al., 2016; Baumann et al., 2021) and plasmalemmal inexcitability (Baumann et al., 2021) as the *mdx* mouse (Fig. 8). These results highlight that, more broadly, a functioning DGC is required to maintain plasmalemmal electrophysiological properties and thus prevent a hypersensitivity to ECCs. Therefore, in addition to all the factors we have already discussed in this review, researchers should also be aware the *mdx* phenotype is evident in dystrophic muscle that is not dystrophin-deficient and appreciate that a collective synergism through each DGC protein is involved in preventing ECC-induced strength loss.

## Closing thoughts

Studying ECC-induced strength loss in dystrophin-deficient skeletal muscle is a common preclinical test to assess disease severity and the efficacy of potential therapies. However, after reading this review, it should be apparent that despite dystrophin-deficient, fast-twitch skeletal muscle being more sensitive to ECC-induced strength loss than wildtype muscle, several caveats must be considered. These include the animal selected, muscle preparation, the ECC protocol utilized, and several intrinsic and extrinsic factors, all of which may impact the mechanisms driving strength loss during and immediately after a bout of ECCs. Moreover, due to the lack of standardized measures when testing ECC-induced strength loss, comparing results across different studies or laboratories may be problematic or at least experiment dependent. We hope that this review provides some clarity for similarities and differences observed in the literature and delivers a backdrop for future investigations. Moreover, we hope that it initiates discussion among researchers on how to optimize ECC physiology protocols to achieve the most robust and accurate preclinical finding that is also translationally relevant.
